# Effects of Superground *Pfaffia glomerata* Leaves on Growth Performance and Immune Function in New Zealand Rabbits

**DOI:** 10.3390/ani15162452

**Published:** 2025-08-21

**Authors:** Yan-Jun Chen, Guang-Zhou Lv, Asim Muhammad, Xin-Yu Zheng, Jia-Hong Xie, Jin-Jun Chen

**Affiliations:** Department of Veterinary Medicine, School of Coastal Agriculture, Guangdong Ocean University, Zhanjiang 524088, China; chenyanjun2@stu.gdou.edu.cn (Y.-J.C.); 13468214365@163.com (G.-Z.L.); asimmuhammad9862@gmail.com (A.M.); 18604068009@stu.gdou.edu.cn (X.-Y.Z.); 18758019771@stu.gdou.edu.cn (J.-H.X.)

**Keywords:** *Pfaffia glomerata* leaves, growth performance, immune function, intestinal flora, New Zealand rabbit

## Abstract

The rabbit industry urgently needs natural alternatives to maintain gut health. This need is growing due to the higher incidence of intestinal problems in antibiotic-free production systems. *Pfaffia glomerata* (Brazilian ginseng) is a medicinal plant rich in bioactive compounds. This study evaluated the effects of dietary inclusion of *P. glomerata* leaf powder on growth performance, immune function, and gut microbiota in New Zealand rabbits. A total of 100 New Zealand rabbits (35 days old) were randomly assigned to five groups (n = 20 each). The groups comprised a blank control group (CON), an immunosuppressed group (CTX), and three treatment groups receiving low (L), medium (M), and high (H) doses of *P. glomerata* leaf powder at 0.5%, 1%, and 2% of the basal diet, respectively. Growth performance was assessed by average daily gain (ADG). Intestinal morphology was evaluated by measuring villus height and crypt depth in the duodenum, jejunum, and ileum. Immune parameters included thymus and spleen weight, serum immunoglobulin levels, and leukocyte counts. Cecal microbiota diversity and composition were analyzed. Compared to CON, *Pfaffia* supplementation improved growth performance: the L and H groups had significantly higher ADG and a lower feed conversion ratio (*p* < 0.05). In intestinal histology, groups L and M had reduced crypt depth in the duodenum (*p* < 0.05), and group L had an increased villus height to crypt depth ratio. In the jejunum and ileum, groups L and H exhibited increased villus height and villus height to crypt depth ratio (*p* < 0.05), indicating an enhanced absorptive surface. In the immunosuppressed model, rabbits in all *Pfaffia* groups showed significantly increased thymus and spleen weight compared to the CTX group (*p* < 0.05) and demonstrated elevated serum immunoglobulins and leukocyte counts (monocytes, lymphocytes, and neutrophils) (*p* < 0.05). *Pfaffia* supplementation also enhanced cecal microbiota diversity and increased the abundance of beneficial bacteria. In summary, dietary *P. glomerata* leaf powder enhanced growth performance, immune organ development, and healthy gut microbiota in growing rabbits. The 0.5% supplementation level (L group) yielded the most consistent benefits. These results suggest that *P. glomerata* is a promising natural feed additive to promote rabbit health and production in antibiotic-free systems.

## Simple Summary

This study aimed to evaluate the effects of supplementing the basal diet of New Zealand rabbits with *Pfaffia glomerata* leaves (family Amaranthaceae, genus *Pfaffia*). We focused on health and production outcomes, including immune function, gut microbiota, and growth performance. The results showed that *P. glomerata* leaves significantly increased the average daily weight gain of the rabbits. It also promoted the development and function of immune organs. Moreover, these leaves improved cecal structure and increased the abundance of beneficial gut bacteria. Among all treatments, the 0.5% supplementation level had the most potent effect on improving growth and immunity.

## 1. Introduction

### 1.1. Application of Plant Additives in Rabbit Farming

Plant additives are feed additives used in the livestock production process after naturally-growing plants have been physically, chemically, or biologically processed. Plant additives can improve rabbit growth performance [1]. On the one hand, they can increase the nutrients in feed, improve digestion and absorption, regulate animal nutrition metabolism, and promote the growth and development of livestock and poultry [2]. On the other hand [3], they can enhance animal immune function, inhibit bacterial growth, and improve animal disease resistance. They can also promote the development of intestinal mucosa [4], improve the diversity of cecal flora [5], and enhance the activity of intestinal enzymes [6]. They also have specific effects in regulating heat stress [7] and in modulating production performance in rabbits [8].

### 1.2. Overview of the Pfaffia glomerata Research

*Pfaffia glomerata* is also known as Brazilian ginseng (Phafophiasan). *P. glomerata* is a perennial herb belonging to the amaranth family, which is mainly distributed in the tropical rainforest area of the Amazon River basin in South America, including Brazil, Ecuador, Paraguay, Peru, and other countries. *P. glomerata* was introduced into China in 2001 and is now cultivated in Jiangxi, Zhejiang, Guangxi, and Guangdong provinces. *P. glomerata* has various biological activities and is an excellent source of exotic Chinese herbal medicine.

There are many chemical components in *P. glomerata*. Ecdysone is the main chemical component isolated and identified from Radix Brasilia. β-ecdysone is the main ecdysone in *P. glomerata*, which exists in the roots, inflorescence, and leaves [9]. Ecdysone content is highest in flowers (0.82%), lower in roots (0.66%), and lower still in stems (0.24%) [10]. In addition, *P. glomerata* contains saponin components [11]. The saponins are usually extracted by mixing water and ethanol [12]. *P. glomerata* also contains flavonoids [13], and its secondary metabolites are inulin polysaccharides [14].

*P. glomerata* has an extreme anti-tumor activity [15]. Studies have shown that it can effectively inhibit the production of melanin without cytotoxicity [16]. It can significantly reduce the volume of ascites in mice with ascites tumors [17]. It also has a specific inhibitory effect on tumor-bearing mice [18]. At the cellular level, *P. glomerata* can effectively induce apoptosis in HepG2 cells. Studies [19] have shown that this extract can reduce cell viability and growth rate without affecting DNA integrity.

### 1.3. The Purpose and Significance of the Study

Currently, China’s rabbit production process is highly dependent on antibiotics. Following the ban on antibiotic use in feed, the survival rate of rabbits in China has decreased significantly. With the development of intensive and large-scale breeding, the demand for antibiotic substitutes in the rabbit breeding process has increased. *P. glomerata* leaves contain a variety of active substances, such as saponins, polysaccharides, and ecdysone [10] to improve animal growth performance and immune function. However, the effects of adding *P. glomerata* leaves to New Zealand the feed of rabbits are unclear.

This study systematically studied the addition of *P. glomerata* leaves into a New Zealand rabbit basic diet and analyzed the effects of dietary supplementation with *P. glomerata* leaves on the health status of New Zealand rabbits in terms of growth performance, immune function, and intestinal flora; this was done to provide an experimental basis for the development and utilization of *P. glomerata* leaf resources and the reduction of antibiotic resistance during the production of New Zealand rabbits.

## 2. Materials and Methods

### 2.1. Experimental Material

*P. glomerata* leaves were collected from Leizhou Peninsula Forest Economic and Technological Innovation Center of Guangdong Ocean University, and identified as *P. glomerata* by the Plant Teaching and Research Department of the Agricultural College of Guangdong University. The nutritional composition table of *P. glomerata* leaves is shown in Table 1. *P. glomerata* leaves were dried at 50 °C for 12 h, and then crushed by an ultrafine mill, and the powder was stored after being sieved through a 300 mesh, with a particle size of approximately 50 μm.

**Table 1:** Nutrition facts table of *Pfaffia glomerata* leaves.

Treatment	Main Ingredients	References
Triterpene saponins	Ginsenoside Re, Rb1, Rg1, Oleanolic acid saponins	[20]
Steroid compounds	β-Ecdysone	[21]
Phenolic compounds	Tannins	[21]
Flavonoids	Quercetin	[22]
Amino acid	Aspartic acid	[22]
Polysaccharide	Polysaccharide	[23]
Neutral detergent fiber	Neutral detergent fiber	[24]
Lignin	Lignin	[24]

We used 100 purebred weaned New Zealand rabbits, aged 35 days, with an average body weight of 496.14 ± 66.4 g. The rabbits included both males and females and exhibited uniform growth conditions. All rabbits were housed in a controlled laboratory setting. The cage allocation was as follows: 5 rabbits were kept in each cage during the first two weeks. From the third week onward, 2 rabbits shared each cage.

The experimental materials comprised: Cyclophosphamide (M17787-5G, Shanghai Meryl Biochemical Technology Co., Ltd., Shanghai, China), Immunoglobulin A (IgA), Immunoglobulin M (IgM), Interleukin-2 (IL-2), Interleukin-6 (IL-6), Tumor necrosis factor-α (TNF-α), Interferon-γ (IFN-γ) detection kit (purchased from Ruiqing Biotechnology Co., Ltd., Nanjing, China). Other reagents were domestic analytical pure.

### 2.2. Experimental Equipment

The main equipment used in this experiment comprised: an ultrafine mill (10L, Guangzhou Remai Machinery Equipment Co., Ltd., Guangzhou, China), herbal medicine dryer (LT-154, Chigo, Foshan, China), feed pellet machine (160 type feed pellet machine, Clay Machinery, Shenyang, China), flake baking machine (MD-II, Jinhua Yidi Medical Equipment Factory, Jinhua, China), rotary microtome (HM325, Thermo Fisher Shanghai Instrument Co., Ltd., Shanghai, China), biological microscope (Eclipse Ci-E, Nikon Instruments Shanghai Co., Ltd., Shanghai, China), electronic balance (AY-120, Shumadzu, Kyoto, Japan), five classification automatic blood cell analyzer (URIT-5180, Guilin Yulite Electronics Group Co., Ltd., Nanning, China), biochemical incubator (SPX-250B-Z, Shanghai Boxun Industrial Co., Ltd. Medical Equipment Factory, Shanghai, China), double water evaporator (Elix100 Millipore Company, Burlington, MA, USA), enzyme label instrument (ELx800, American Berton Instrument Co., Ltd., Vermont, VT, USA), and horizontal centrifuges (Rotina 35 R Hettich, Kirchlengern, Germany).

### 2.3. Experimental Methods

#### 2.3.1. Experimental Design

In this study, a single-factor experimental design was adopted. A total of 5 experimental treatment groups were set up: the CON group, were fed only the basal diet, while the CTX group, were fed with the basal diet and injected daily with 50 mg/kg of cyclophosphamide in the gluteal muscle during the last week of the trial. The experimental groups were the low dose group (group L), medium dose group (group M), and high dose group (group H), which were fed the basal diet supplemented with 0.5%, 1%, and 2% superfine powder of *P. glomerata* leaves, respectively. Rabbits were injected daily in the gluteal muscle with 50 mg/kg cyclophosphamide in the last week of the experiment. Each group consisted of 4 replicates with 5 rabbits per replicate.

#### 2.3.2. Feeding Management

The trial was conducted at the Forest Economic Science and Technology Innovation Center of the Leizhou Peninsula, Guangdong Province, from August to October 2023. Rabbits had ad libitum access to feed and water during the experimental period. Animal experiment protocols were conducted by the ethical guidelines and approved plans of the Animal Ethics Committee of Guangdong Ocean University (IACUC No. GDOU-NXY-2023-039-410). All experiments were performed according to relevant guidelines and regulations. Our reporting of research involving animals follows the recommendations of the ARRIVE guidelines. All rabbits were maintained separately in galvanized wire pens (35 × 35 × 60 cm). The basal diet composition is shown in Table 2. The experiment included a 7-day adaptation period, followed by a 63-day formal trial. Before introducing the animals, the empty cages were thoroughly disinfected using spray disinfection. After the rabbits were placed in the cages, cleaning and disinfection were performed once or twice a week. At the end of the trial period, the rabbits were 105 days old. The lighting plan during the experiment was as follows: 1 month before the experiment, the rabbits were placed under natural light. Starting from the 23rd day of the experiment, in addition to natural light exposure, the lights were turned on for 1 h before feeding every night.

#### 2.3.3. Production Performance Index Measurement

On the 63rd day of the experiment, the New Zealand rabbits were deprived of feed and kept hydrated for 1 day. They were weighed on an empty stomach at 8:00 the next day to calculate the total feed intake, the number of dead rabbits, the average daily feed intake, the average daily gain, and the feed conversion ratio (in the case of death during the experiment, the total intake of dead rabbits should be recorded promptly).

Calculation method:

Average daily feed intake (g) = feed intake/(test days × number of rabbits per group)

Average daily gain (g) = (end of trial weight-beginning of trial weight)/trial days

Feed conversion ratio (%) = Average daily feed intake/average daily gain

#### 2.3.4. Intestinal Development Measurement

On the 63rd day of the experiment, 5 New Zealand rabbits with similar body weights were selected from each group and sampled after euthanasia. Subsequently, the duodenum, jejunum, and ileum were removed. The intestinal chyme was first rinsed with normal saline, then fixed and preserved with 4% paraformaldehyde, and then HE staining and paraffin sectioning was performed.

#### 2.3.5. Immune Organ Index Measurement

On the 63rd day of the experiment, the experimental rabbits were only fed, given water, and weighed. Five rabbits were selected from each group and sampled after euthanasia. After death, the spleen, thymus, and sac of the experimental rabbits were collected from the chest and abdominal cavity, and washed with normal saline. Other tissues were removed, and the removed immune organs were weighed using an electronic balance. After weighing, the immune index of the immune organs was calculated according to the following formula. Immune organ index = immune organ mass (g)/rabbit body weight (g) × 100%.

#### 2.3.6. Determination of Blood Indexes

The day after the final administration of medicine, 0.5 mL of venous blood was collected into an EDTA collection vessel using ear vein blood collection. The changes in white blood cells (WBC), lymphocytes (LYM), monocytes (MON), neutrophils (NEU), eosinophils (EOS), basophils (BASO), red blood cells (RBC), hemoglobin (HGB), and platelets (PLT) in the blood were detected using an automatic blood cell analyzer.

#### 2.3.7. Determination of Immunoglobulin and Immune Factor in Serum

Eight samples were selected for each group. On the day after the last administration, 2 mL of blood was collected from the ear vein in the collection vessel and transferred to a 2 mL clean centrifuge tube at 3000 r/min for 10 min, and the upper serum was collected. The changes in the contents of immunoglobulins and immune factors in the blood serum were detected by enzyme-linked immunosorbent assay.

#### 2.3.8. Intestinal Flora 16S rDNA Sequencing

On the 63rd day of the experiment, two rabbits were selected from each replicate, and eight rabbits in each group were collected. Two grams of cecal contents were collected in 2 mL test tubes and, immediately after collection, temporarily placed in liquid nitrogen, and then stored in the refrigerator at −80 °C until use.

T Guide S96 was used to extract genomic DNA from the cecum to complete the extraction of nucleic acids, and Qubit was used to detect the concentration of extracted nucleic acids quantitatively. The PCR products were amplified according to the detection method, and their integrity was tested by electrophoresis on a 1.8% agarose gel.

The target region PCR system was constructed for qualified samples, product purification and PCR amplification were performed, non-specific bands were removed, and the target DNA was recovered using a DNA purification and recovery kit. A DNA construction kit was used to construct the library, and the Illumina Novaseq 6000 (Illumina, San Diego, CA, USA) was used for on-machine sequencing of the constructed library.

#### 2.3.9. Data Processing and Analysis

The sequenced Raw Reads were filtered using Trim Momatic v0.33 software. Then, cutadapt1.9.1 software was used to identify and remove primer sequences, and Clean Reads without primer sequences were obtained. The dada2 method in QIIME2 2020.6 was used for denoising, the double-ended sequences were spliced, and the chimeric sequences were removed to obtain the final effective data (Non-chimeric Reads). Information analysis content was divided into feature (OTUs, ASVs), diversity analysis, difference analysis, correlation analysis, and function prediction analysis.

The results of each group were analyzed by using GraphPad Prism 9.5 software. The measurement data of each group all conform to the normal distribution. Data are expressed as mean ± standard deviation. After analysis using one-way analysis of variance, further multiple comparisons were conducted with the control group and the immunosuppression group. For statistical analysis, *p* < 0.05 indicates that there is a statistically significant difference between the two groups.

## 3. Results

### 3.1. Effects of Pfaffia glomerata Leaves on Production Performance 

As shown in Table 3, compared with the control group, the average daily feed intake of New Zealand rabbits in the L group and the H group was significantly reduced, and the difference was statistically significant (*p* < 0.05). At the same time, there was no statistically significant difference in the M group (*p* > 0.05). The average daily weight gain of the *P. glomerata* leaf addition group was higher than that of the control group. In this measure, the L group and the M group were significantly higher than the control group (*p* < 0.05), but the increase in the H group was not significant (*p* > 0.05). The feed conversion ratio was significantly reduced in the L group and the H group (*p* < 0.05), while the M group showed no significant decrease (*p* > 0.05).

### 3.2. Effects of Pfaffia glomerata Leaves on Intestinal Function 

As shown in Table 4, adding *P. glomerata* leaves to the diet can improve the intestinal structure of New Zealand rabbits. Compared with the control group, the height of duodenal villi in rabbits in group L and group H increased, but there was no significant effect (*p* > 0.05). The crypt depth in group L and group M was significantly decreased (*P* < 0.05), while there was no statistically significant difference in group H (*p* > 0.05). In group L, there was a significant increase in the villus height to crypt depth ratio (*p* < 0.05), while the villus height to crypt depth ratio was increased in groups M and H, but not significantly (*p* > 0.05).

Compared with the control group, the villus height of the jejunum in both group L and group H was significantly increased (*p* < 0.05). Compared with the control group, the villus height to crypt depth ratio of both group L and group H was significantly increased (*p* < 0.05).

Compared with the control group, the intestinal villus height in the ileum increased in all *P. glomerata* leaf addition groups, and there was a significant increase in groups L and H (*p* < 0.05). However, no significant difference was observed in group M (*p* > 0.05). There was no significant difference in the crypt depth of the ileum in the added group (*p* > 0.05). The villus height to crypt depth ratio in group L was significantly increased (*p* < 0.05), but there was no significant difference between groups M and H (*p* > 0.05).

### 3.3. Effects of Pfaffia glomerata Leaves on Immune Organ Indexes

As shown in Table 5, compared with the CON group, the immune organ quality and immune organ index of the CTX group were decreased, except that the thymus index was not significantly decreased (*p* > 0.05), and other groups were significantly decreased (*p* < 0.05). Compared with the CTX group, the thymus mass and saccus mass of the *P. glomerata* leaf addition group were significantly increased (*p* < 0.05), the thymus index of groups M and H were significantly increased (*p* < 0.05), the thymus index of group L was increased but not significantly (*p* > 0.05), and the saccus index in groups L and M were significantly increased (*p* < 0.05). In group H, it increased but not significantly (*p* > 0.05), and there were no significant changes in spleen mass and spleen index in groups supplemented with *P. glomerata* leaves (*p* > 0.05).

### 3.4. Effects of Pfaffia glomerata Leaves on Hematological

**Figure 1:**
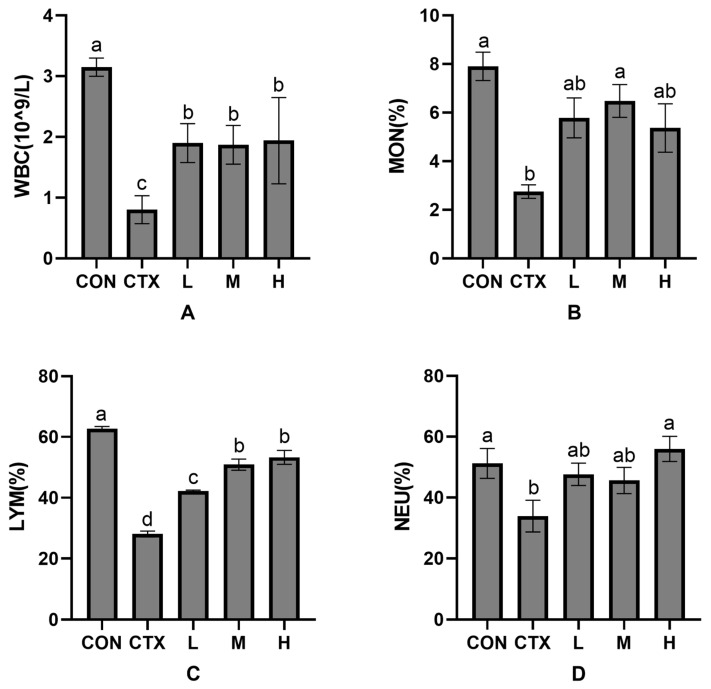
Effects of *Pfaffia glomerata* leaves on serological indicators (WBC, MON, LYM and NEU) in New Zealand rabbits. (**A**) The influence of *P. glomerata* leaves on white blood cells; (**B**) The influence of *P. glomerata* leaves on monocytes; (**C**) The influence of *P. glomerata* leaves on lymphocytes; (**D**) The influence of *P. glomerata* leaves on neutrophils. The results are expressed as mean ± SD, n = 8. Different letters on the inter-digit scale indicate significant differences (*p* < 0.05), lowercase letters indicate significance level a = 0.01, and the same letter or no letter indicates no significant difference (*p* > 0.05).

As can be seen from Figure 1, compared with the CON group, white blood cells (WBC), monocytes (MON), lymphocytes (LYM), and neutrophils (NEU) in the CTX group were significantly decreased (*p* < 0.05). Compared with the CTX group (Figure 1A), the WBC count significantly increased in all *P. glomerata*-supplemented groups (*p* < 0.05), and there were significant differences among all groups (*p* < 0.05). MON (Figure 1B) were significantly increased in all groups supplemented with *P. glomerata* leaves (*p* < 0.05), but no significant difference was found among all groups (*p* > 0.05). LYM (Figure 1C) in all groups supplemented with *P. glomerata* leaves were significantly increased (*p* < 0.05), but there was no significant difference among all groups (*p* > 0.05). NEU (Figure 1D) in groups L and M showed an increase, but this was not significant (*p* > 0.05), while those in group H had a significant increase (*p* < 0.05). Therefore, group M in group MON had the best effect, and group H in group NEU had the best effect (Figure 1B,D).

**Figure 2:**
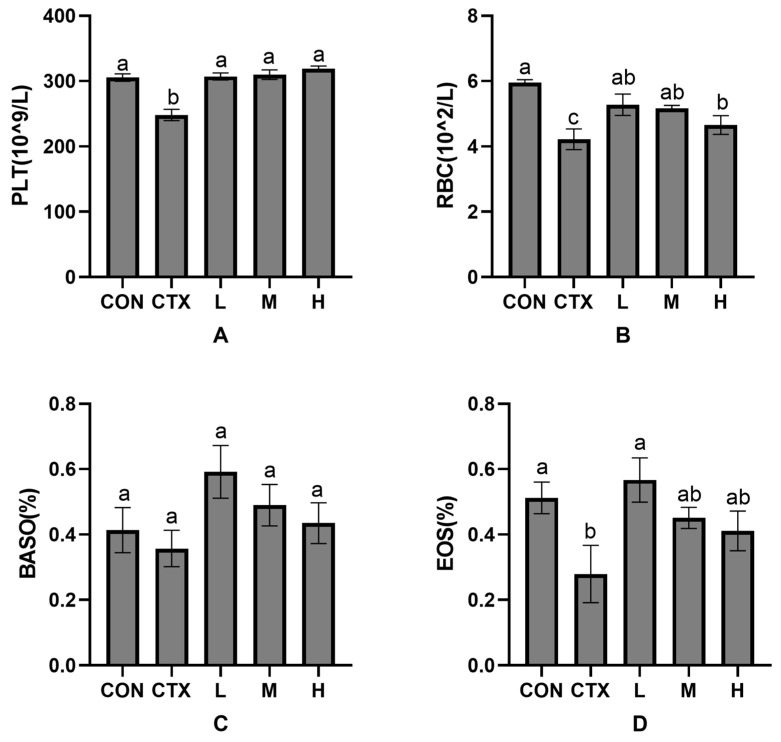
Effects of *Pfaffia glomerata* leaves on serological indicators (PLT, RBC, BASO and EOS) in New Zealand rabbits. (**A**) The influence of *P. glomerata* leaves on platelets; (**B**) The influence of *P. glomerata* leaves on red blood cells; (**C**) The influence of *P. glomerata* leaves on basophils; (**D**) The influence of *P. glomerata* leaves on eosinophils. The results are expressed as mean ± SD, n = 8. Different letters on the inter-digit scale indicate significant differences (*p* < 0.05), lowercase letters indicate significance level a = 0.01, and the same letter or no letter indicates no significant difference (*p* > 0.05).

As shown in Figure 2, compared with the CON group, red blood cells (RBC) and eosinophils (EOS) in the CTX group were significantly decreased (*p* < 0.05). In contrast, platelets (PLT) and basophils (BASO) were decreased but not significantly (*p* > 0.05). Compared with the CTX group (Figure 2A), PLT were significantly increased in both groups (*p* < 0.05), but no significant difference was found among all groups (*p* > 0.05). RBC (Figure 2B) in the *P. glomerata* leaf addition group were significantly increased (*p* < 0.05), but there was no significant difference among all groups (*p* > 0.05). BASO (Figure 2C) were increased but not significantly (*p* > 0.05) in the *P. glomerata* leaves addition group, and there was no significant difference among all groups (*p* > 0.05). EOS (Figure 2D) in group L were significantly increased (*p* < 0.05), while those in groups M and H was increased but not significantly (*p* > 0.05).

### 3.5. Effects of Pfaffia glomerata Leaves on Immunoglobulin Contents 

Compared with the CON group, IgM and IgA levels were significantly decreased in the CTX group (Table 6). According to the changes in IgM, compared with the CON group, the *P. glomerata* leaf addition group showed a significant increase in all three groups (*p* < 0.05). Compared with the CTX group, all three groups showed significant increases (*p* < 0.05), and intra-group comparison showed that group H was significantly increased compared with groups L and M (*p* < 0.05). Compared to the CON group, there was no significant difference in IgA in group M (*p* > 0.05), an increase in group L (*p* < 0.05), a significant increase in group H (*p* < 0.05), and a comparison within groups showed that there was a significant increase in group H (*p* < 0.05). The results of immunoglobulin showed that a 2% dose of *P. glomerata* leaves showed the most significant effect.

### 3.6. Effects of Pfaffia glomerata Leaf Addition on Serum Immune Factors 

**Figure 3:**
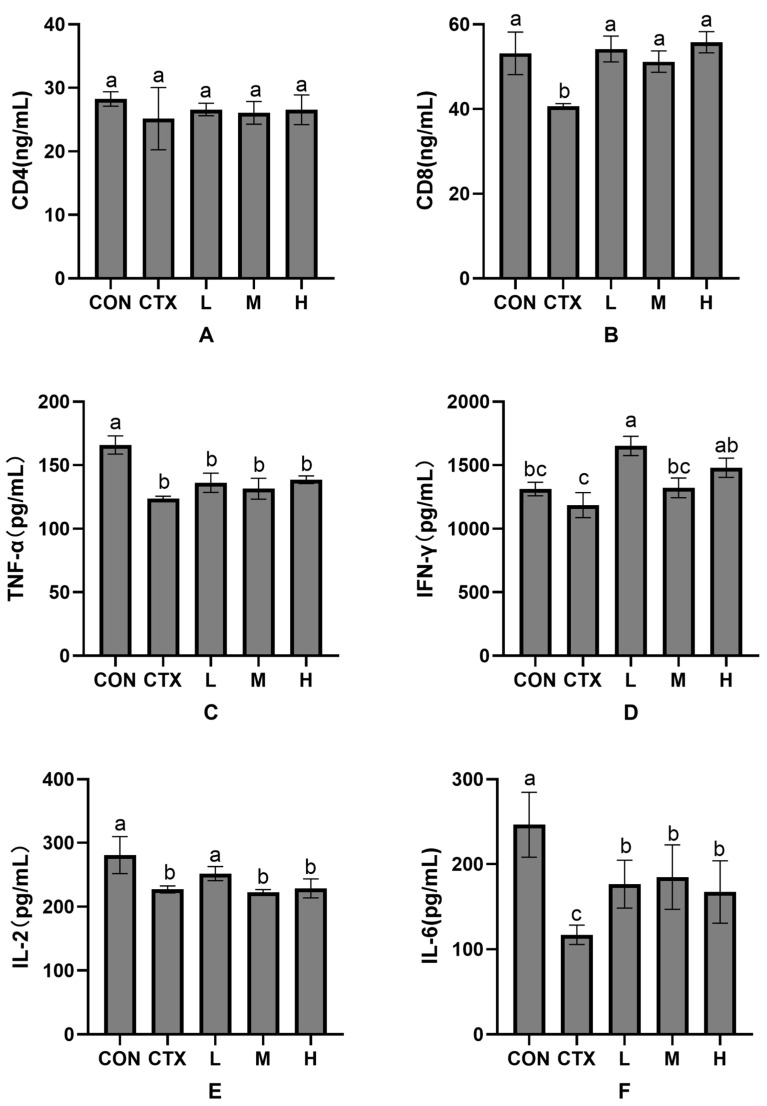
Effects of *Pfaffia glomerata* leaves on serum immune factors (CD4, CD8, TNF-α, INF-γ, IL-2, and IL-6) in New Zealand rabbits. (**A**) The influence of *P. glomerata* leaves on CD4; (**B**) The influence of *P. glomerata* leaves on CD8; (**C**) The influence of *P. glomerata* leaves on TNF-α; (**D**) The influence of *P. glomerata* leaves on IFN-γ; (**E**) The influence of *P. glomerata* leaves on IL-2; (**F**) The influence of *P. glomerata* leaves on IL-6. The results are expressed as mean ±SD, n = 8. Different letters on the inter-digit scale indicate significant differences (*p* < 0.05), lowercase letters indicate significance level a = 0.01, and the same letter or no letter indicates no significant difference (*p* > 0.05).

As shown in Figure 3, compared with the CON group, the levels of CD4 and IFN-γ were decreased but not significantly (*p* > 0.05), while the levels of CD8, TNF-α, IL-2 and IL-6 were significantly decreased in the CTX group (*p* < 0.05). There was no significant difference in CD4 level between the *P. glomerata* leaf addition group and the CON and CTX groups (*p* > 0.05) (Figure 3A). There was no significant difference in CD8 content between the *P. glomerata* leaf addition group and the CON group (*p* > 0.05). At the same time, CD8 was significantly increased compared with the CTX group (*p* < 0.05) (Figure 3B). Compared with the CTX group, the content of TNF-α was increased but not significantly (*p* > 0.05) (Figure 3C). Compared with the CTX group, IFN-γ contents in groups L and H were significantly increased (*p* < 0.05), and IFN-γ content in group M was increased but not significantly (*p* > 0.05) (Figure 3D). Compared with the CTX group, the content of IL-2 in group L was significantly increased (*p* < 0.05), the content of IL-2 in groups M and H was increased but not significantly (*p* > 0.05) (Figure 3E), and the content of IL-6 in group H was significantly increased compared with that in the CTX group (Figure 3F).

### 3.7. OTU Analysis of Rabbit Cecum Microorganisms

**Figure 4:**
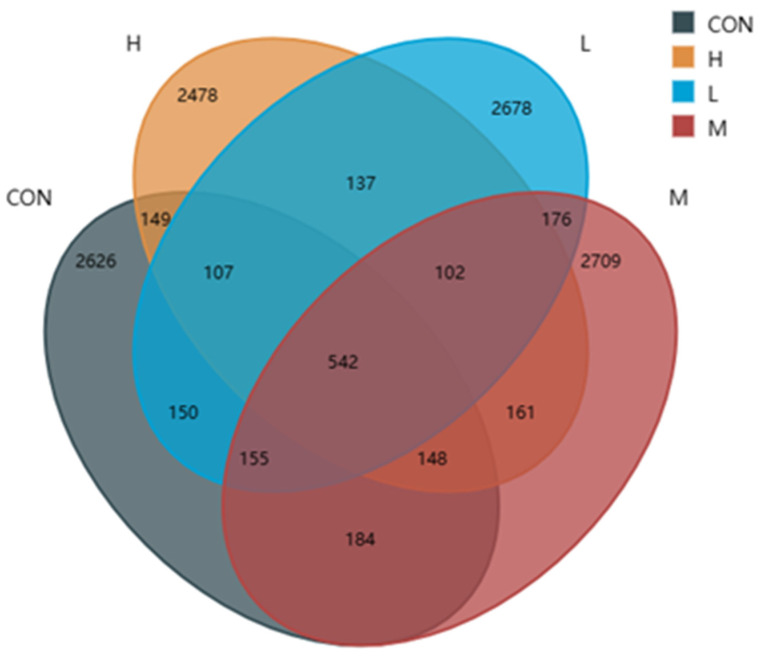
Venn diagram of cecal microflora OTUs. CON was the control group; L, M and H were 0.5%, 1% and 2% *P. glomerata* leaf addition groups, respectively.

The four groups had a total of 9860 OTU_S_, among which the control group had 2994 OTUs, the L group had 3456 OTUs, the M group had 2874 OTUs, and the H group had 3315 OTUs (Figure 4). There were 150 OTUs in the L group, 184 in the M group, 149 in the H group, and 542 OTU_S_ in the four groups. There were 2626 OTUs in the blank group, 2678 OTUs in the L group, 2478 OTUs in the M group, and 2478 OTUs in the H group. The results showed that the total number of cecal microbiota OTUs in Group L and Group H was significantly increased compared with that in the control group.

### 3.8. Analysis of Microbial Alpha Diversity in the Cecum

**Figure 5:**
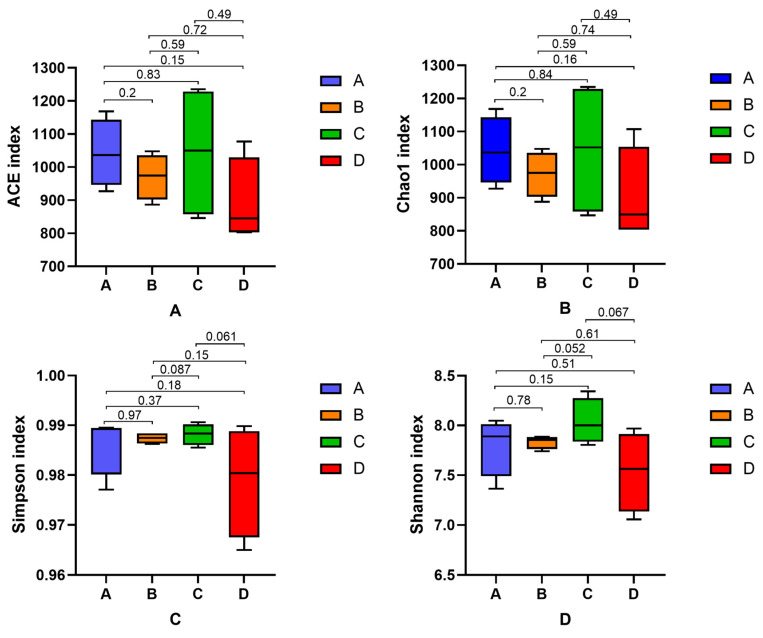
Analysis of microbial Alpha diversity in the cecum. (**A**) ACE index; (**B**) Chao1 index; (**C**) Simpson index; (**D**) Shannon index.

Compared with the blank group (Figure 5A,B), the ACE index and Chao1 index of cecal colonies in each group were increased after feeding *P. glomerata* leaves. However, only the low dose group showed a significant difference (*p* < 0.05). The Simpson index and Shannon index in the added group showed increases compared with the blank group, but there was no significant difference (*p* > 0.05) (Figure 5C,D). The addition of *P. glomerata* leaves increased the diversity of the cecum microbial community in New Zealand rabbits.

**Figure 6:**
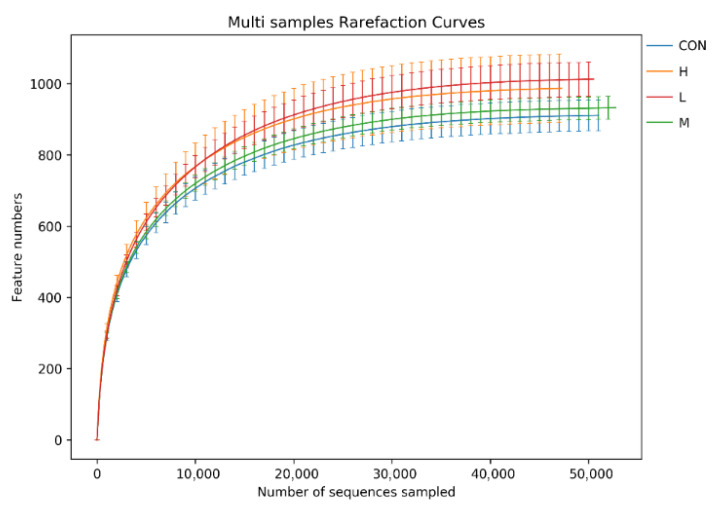
Sample dilution curve. CON was the control group; L, M and H were 0.5%, 1% and 2% *P. glomerata* leaf addition groups, respectively.

The Rarefaction Curve is the basis for judging the sequencing quantity of each sample, and can also indirectly reflect the species abundance of cecum contents. The number of species increased sharply with the increase in the number of sequencing lines. Eventually, it stabilized, indicating that the cecum content samples were sequenced adequately. The microbial species in the samples showed no significant increase with the increase in the number of sequencing lines, which could be further analyzed (Figure 6). Therefore, the dilution curve is the basis for judging the sequencing quantity of each sample, and can also indirectly reflect the species abundance of cecal contents. The species richness of added components in the leaves of *P. glomerata* was higher than that in the blank group.

### 3.9. Analysis of Beta Diversity of Cecum Microorganisms

**Figure 7:**
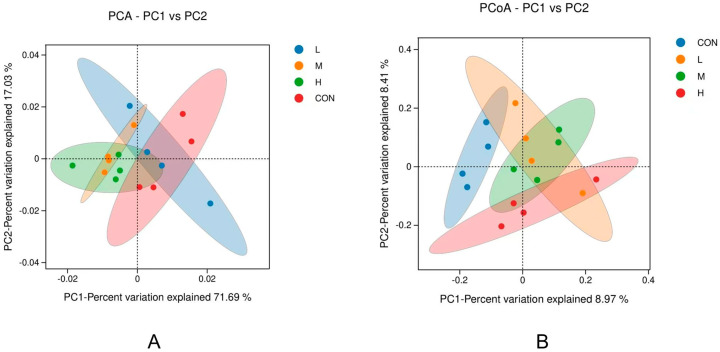
Analysis of Beta diversity of cecum microorganisms. (**A**) PCA analysis; (**B**) PCOA analysis.

The contribution rate of principal component 1 was 71.69%, and the contribution rate of principal component 2 was 17.03%; in Figure 7, the red is the 95% confidence ellipse of the control group. The distance between samples in the PCA (Figure 7A) diagram is related to their community differences, and the smaller the difference, the closer the distance. It can be found that there is little overlap between group M and group H and the blank group, indicating that the biome of these two groups is significantly different from that of the blank group, and group L has more overlap with the blank group, indicating that there is no significant difference from the blank group. It was also found that the difference in microbial community between group M and group H was slight. According to the results of PCOA (Figure 7B), the contribution rates of principal components 1 and 2 were 8.97% and 8.41%, respectively. The 95% confidence ellipses of the H and M groups did not overlap with those of the blank group, group L partially overlapped, and there were overlaps between groups with added *P. glomerata* leaves. These results indicated that the microbial communities of groups M and H were significantly different from those of the blank group, and there was no significant difference between groups supplemented with *P. glomerata* leaf.

### 3.10. Abundance Analysis of the Cecum Microbial Community

**Figure 8:**
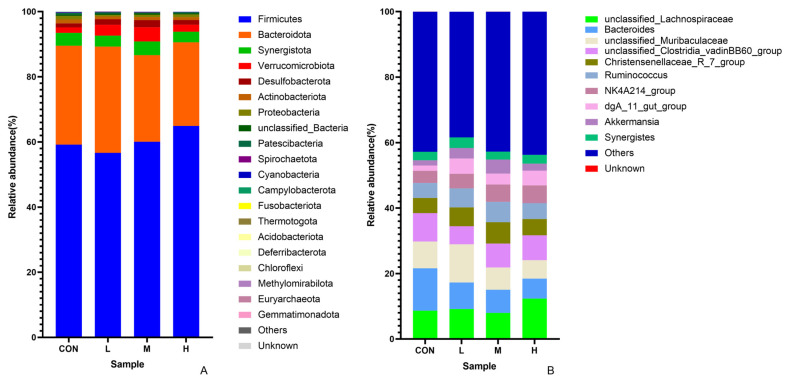
Abundance analysis of cecum microbial community. (**A**) PCA analysis; (**B**) PCOA analysis.

The top ten dominant intestinal flora in the cecum of New Zealand rabbits were selected at the phylum level, and the dominant flora types in each group are shown in Figure 8A. The dominant bacteria are *Firmicutes*, *Bacteroidota*, *Synergistota*, *Verrucomicrobiota*, *Desulfobacterota*, *Actinobacteriota*, *Proteobacteria*, *unclassified bacteria*, and *Patescibacteria*. The top four dominant bacteria accounted for more than 90% of the total. Compared with the control group, the abundance increased, and the Bacteroidetes abundance decreased in the medium dose and high dose groups. The abundance of *Verrucomicrobacteria* increased, while the abundance of *Proteobacteria* and prion bacteria decreased in the *P. glomerata* leaf addition group. It is worth noting that the sum of the proportion of the top five dominant bacteria in the *P. glomerata* leaf addition group was higher than that in the control group. Hence, the *P. glomerata* leaf addition increased the abundance of the main dominant bacteria in the cecum of New Zealand rabbits to a certain extent.

Through further analysis of the microbial community level in the cecum, it was found (Figure 8B) that the dominant bacterial genera in each group were unclassified *Lachnospiraceae*, *Bacteroides*, *unclassified Muribaculaceae*, *unclassified_Clostridia_vadinBB60_group* and *Christensenellaceae_R_7_group*, *Ruminococcus*, *NK4A214_group*, *dgA_11_gut_group*, *Akkermansia* and *Synergistes*. Compared with the control group, the abundance of Bacteroides in the cecum of New Zealand rabbits was decreased in each *P*. *glomerata* supplementation group. In contrast, the abundance of *Helicobacter*, *Ruminococcus* and *Ackermansia* was increased. The abundance of the unclassified R-7group of *Christensenaceae* and the NK4A214 group of *Ruminococcus* increased in the medium and high dose groups (Table 7 and Table 8).

## 4. Discussion

### 4.1. Effects of Pfaffia glomerata Leaves on Production Performance 

Research on the main chemical components of the leaves of the *P. glomerata* shows that the main active components of the stems and leaves of the *P. glomerata* are similar, including chemical components such as β-ecdysone and flavonoids [25], suggesting that the leaves of *P. glomerata* also have similar biological activities. Average daily gain and feed conversion rate are important in livestock production. The L and M groups of *P. glomerata* leaves could significantly increase the average daily weight gain of New Zealand rabbits. The L, M and H groups could all reduce the feed conversion rate. However, the average daily weight gain of the control group was very low. We speculate that adding a small amount of *P. glomerata* leaves to the basic diet might promote the feeding of New Zealand rabbits. However, the leaves of the *P. glomerata* are bitter. Adding a large amount of *P. glomerata* leaves to the diet may lead to a decrease in rabbits’ feed intake. The results of group H are consistent with this theory. *P. glomerata* is rich in ginsenosides, triterpenoid saponins, and inulin polysaccharides, which can regulate glucose and lipid metabolism [26], improve the intestinal physical barrier [27], and enhance intestinal digestive function [28]. From this, we speculate that the effective improvement of the growth performance of New Zealand rabbits by *P. glomerata* leaves might be due to the inulin polysaccharides and saponins in the leaves.

### 4.2. Effects of Pfaffia glomerata Leaves on Intestinal Morphology

The small intestine is the primary site where the body absorbs nutrients, and it is mainly divided into three parts: the duodenum, jejunum, and ileum. Intestinal villi and recesses are special structures of the intestine and are important indicators of the digestion and absorption capacity of the small intestine [25]. In this study, we found that low and high doses of *P. glomerata* leaves improved the small intestine structure, increased the length of the small intestine villi, reduced the depth of crypts, and increased the villus height to crypt depth ratio. Studies have found that adding algal supplements [29] or inulin and flavonoids [30] to the basal diet can effectively increase the intestinal villi length of rabbits and maintain the integrity of the intestinal structure, which is consistent with the results of this experiment. Some alkaloids can improve the intestinal structure of animals. Sanguinarine can protect against intestinal injury caused by radiation by down-regulating the HMGB1/TLR4 pathway and reducing pro-inflammatory cytokines [31]. Studies have shown that oleanolic acid can relieve colitis [32]. The leaves of *P. glomerata* contain active components, such as alkaloids and oleanolic acid [22]. Therefore, we speculate that the improvement effect of *P. glomerata* leaves on the intestinal structure of New Zealand rabbits may be related to the presence of alkaloids or oleanolic acid.

### 4.3. Pfaffia glomerata Leaf Can Improve the Immune Organ Index 

The thymus is the central immune organ of the body and is mainly responsible for transporting immune cells to the peripheral immune organs [33]. The spleen is the largest peripheral immune organ in the body and plays a role in storing immune cells for the immune response. Simultaneously, the spleen is necessary for antibody-mediated immune enhancement [34]. In addition, the small round sac, also known as the lymphatic balloon, is formed at the junction of the ileum and cecum [35]. As a unique peripheral organ in rabbits, the round sacs have both digestive and endocrine functions. The immune organ index is one of the main criteria for immune organ development and function. In this study, we found that cyclophosphamide would inhibit the development of immune organs in New Zealand rabbits, thereby leading to a decrease in immune organ indices. The addition of *P. glomerata* leaves can reduce the downward trend of the thymus index caused by CTX, with the medium dose of *P. glomerata* leaves having the best effect. In addition, compared with the CTX group, the index of the sacculus rotundus of the *P. glomerata* leaves in the medium dose group was significantly increased, indicating that the medium dose can promote the growth and development of the thymus and sacculus rotundus of New Zealand rabbits, increase the index of immune organs, and thereby achieve the effect of enhancing their immunity.

### 4.4. Effects of Pfaffia glomerata Leaves on Blood Indexes 

Blood is an important medium for the body to exert immune functions, and all types of immune cells are transported to the designated parts through blood. White blood cells are the second line of defense of the body. Their primary function is to engulf pathogens. They can also engulf pathogens outside the blood vessels through capillary malformations [36]. Neutrophils contain a variety of lysozymes that digest and phagocytose foreign bodies and bacteria [37]. Basophilic granulocytes release histamine, platelet-activating factor, basophilic chemotactic factor and other bioactive mediators that cause hypersensitivity reactions after antigen stimulation [38]. Eosinophils can limit hypersensitivity in the body and have a synergistic effect with basophils [39]. Lymphocytes are the primary functional cells in white blood cells, with a strong phagocytic ability. Red blood cells transport O_2_ and CO_2_ into the blood, and participate in the immune response. Studies have found that red blood cells can clean up immune complexes, regulate cytokines, enhance immune response and regulate the role of cytokines [40]. Platelets are primarily involved in blood coagulation. In addition, platelets can participate in immune regulation by expressing Pattern Recognition Receptor (PRR) [41]. These cells are involved in the immune response of the body, and their changes are closely related to the immune function of the body. If the cellular levels of this series decline, it is indicated as immunosuppression; conversely, it indicates immune enhancement. Some studies have found that CTX, as an immunosuppressant, can reduce the number of white and red blood cells in the blood [42], and the trend change in this study is the same. In this study, it was found that various blood cell parameters in the immunosuppressive group were significantly reduced. The addition of *P. glomerata* leaf improved the decrease in the number of various blood cells caused by cyclophosphamide, indicating that *P. glomerata* leaf could improve the immune function decline caused by cyclophosphamide in New Zealand rabbits.

### 4.5. Pfaffia glomerata Leaves Can Increase the Serum Immunoglobulin Level 

Immunoglobulin is a protein secreted by plasma cells after antigen stimulation of B cells, and is mainly involved in humoral immunity. IgM can combine with complement to dissolve bacteria and neutralize viruses. IgA is abundant in mucous tissue and body fluids, and has a defense effect against pathogen invasion. In this study, cyclophosphamide caused a decrease in serum immunoglobulin levels. IgM and IgA levels in the high dose experimental group were significantly increased, indicating that high dose *P. glomerata* leaf supplementation can improve the level of immunoglobulin in New Zealand rabbits. Some researchers have found [43] that the *P. glomerata* compound increased IgM and IgA content in immunosuppressed mice, which was consistent with the results of this experiment. In addition, flavonoid extracts of sea buckthorn increased the levels of immunoglobulin IgA and IgM in meat rabbits [44], which was consistent with the increase of immunoglobulin in the high dose group of *P. glomerata* leaves in this experiment. From this, the increase in immunoglobulin levels in New Zealand rabbits may be related to the flavonoid saponins contained in the leaves of the *P. glomerata*.

### 4.6. Pfaffia glomerata Leaves Can Increase the Content of Serum Immune Factors

CD4 and CD8 are two surface glycoproteins involved in T cell antigen recognition and activation, and their expression is closely related to T cell antigen specificity. T cells are divided into CD4+T cells and CD8+T cells, according to the type of CD molecule expressed on the surface of T cells. The results indicated that cyclophosphamide could cause immunosuppression in New Zealand rabbits, further leading to a decrease in the content of CD8. The addition of *P. glomerata* leaves had no significant effect on the content of serum CD4 molecules, but it could effectively increase the content of CD8 inhibited by cyclophosphamide. From this, we infer that adding *P. glomerata* leaves can effectively promote the expression of CD8 molecules, thereby increasing the content of CD8 + T cells and enhancing the antiviral ability and immune function of New Zealand rabbits.

Tumor necrosis factor-α (TNF-α) is produced by macrophages and monocytes [45]. When antigens stimulate macrophages, they secrete TNF-α, which participates in the body’s immune response. Interleukin is the main cytokine in serum, which can regulate the inflammatory response of the body and participate in cellular immunity and humoral immunity. It was found that the addition of low and high doses of *P. glomerata* leaves improved the abnormal cytokine levels caused by cyclophosphamide, played a positive regulatory role, and enhanced the immune function of New Zealand rabbits. Studies have shown that [46] bamboo leaf flavonoids can effectively improve the concentrations of IL-2 and IFN-γ in serum and the gene expression of IL-2 and IFN-γ in the spleen. Therefore, it can be inferred that the increase in cytokine levels in the leaves of the control group may be related to the flavonoids present in the leaves.

### 4.7. Pfaffia glomerata Leaves Can Increase the Abundance of the Dominant Intestinal Microbiota in the Cecum

The balance between various microbial communities is an important guarantee of animal health. In meat rabbits, digestion and absorption the structure of the cecum microbial community are closely related to healthy production. A Venn map can directly show the degree of similarity and difference of gut microbes among different groups. The results of this experiment showed that the total OTU of cecal microbes in the 0.5% and 2% groups was significantly higher than that in the control group. Adding *P. glomerata* leaves can improve the diversity of the intestinal flora in New Zealand rabbits.

Intestinal flora Alpha diversity is an evaluation index of intestinal microbial community diversity and richness. Alpha diversity is represented by Chao1, ACE, and the Shannon and Simpson indices. The Chao1 index and ACE index represent the richness of microbial communities, while the Shannon index and the Simpson index represent species diversity. This study found that the addition of *P. glomerata* leaves could increase the Chao1 index, ACE index, Shannon index and Simpson index of New Zealand rabbits. The addition of *P. glomerata* leaves increased the differences in the intestinal flora of New Zealand rabbits to a certain extent. Peng et al. [47] found that anthocyanin flavonoids could significantly increase the Chao1 index and ACE index of the intestinal microbial community in mice, and PCA results in the anthocyanin group did not overlap with those in the control group, increasing the α diversity and β diversity of mice. Therefore, we speculate that the regulatory effect of *P. glomerata* leaves on the intestinal flora may be related to the flavonoids and polysaccharides they contain.

The thick-walled bacteria phylum [48] can maintain intestinal homeostasis through the dietary fiber–thick-walled bacteria–host axis pathway. Bacteroides can participate in intestinal sugar metabolism [49], and *verrucobacteria* can participate in the decomposition of small molecules of fiber polysaccharides, which can also enhance intestinal immune function. The results of this experiment showed that the addition of *P. glomerata* leaves changed the microflora distribution in the caecum of New Zealand rabbits at the phylum level and genus level. The addition of *P. glomerata* leaves reduces the abundance of *Proteobacteria* and actinomycetes, which contain a variety of pathogenic bacteria [50] and are harmful to animal health. It can be inferred that *P. glomerata* leaves can promote the proliferation of beneficial bacteria in the intestinal tract of New Zealand rabbits, inhibit the number of some harmful bacteria, and maintain intestinal health. *Helicobacter* can use glucose and intermediates, such as acetate and lactic acid, to form butyrate by fermentation, and some studies have found that its metabolites can prevent rectal cancer [51]. *Ruminococcus* and *NK4A214_group* belong to the *Ruminococcus* family and can decompose cellulose in the cecum. It can be inferred that the addition of *P. glomerata* leaves will increase the abundance of beneficial bacteria in part of the cecum, promote the decomposition of cellulose in the intestine, improve intestinal sugar metabolism, and thus improve the growth performance of New Zealand rabbits. The above conclusion was drawn in this study through 16S rDNA sequencing analysis. Further research is needed to explain the comprehensive impact of *P. glomerata* leaves on the intestinal flora of New Zealand rabbits, if more evidence is required.

## 5. Conclusions

Dietary supplementation with ground *P. glomerata* leaves at doses ranging from 0.5% up to 2% effectively enhanced the growth performance and immune function of New Zealand rabbits. Among these dietary doses, the addition of *P. glomerata* leaves at 0.5% had the best effect in increasing average daily weight gain, improving intestinal mucosa results, and optimizing the structure of intestinal flora.

In conclusion, the addition of 0.5% *P. glomerata* leaf can promote the growth of New Zealand rabbits, enhance their immune function, improve their intestinal mucosal structure, optimize their intestinal flora structure, and help to ensure their healthy breeding.

## Figures and Tables

**Table 2:** Ingredient and nutrient composition of the basic diet.

Ingredients	Content (%)
Corn	25
Wheat	22
Soybean meal	15
Alfalfa meal	35
CaPO_4_	1
NaCl	0.5
Vitamins	0.5
Premix	1
Total	100
Digestible energy (MJ/kg)	11.37
Crude protein	15
Crude fiber	25
Ca	0.82
P	0.46

**Table 3:** Effects of adding *Pfaffia glomerata* leaves to the basic diet on the growth performance of New Zealand rabbits.

Treatment	Groups			
	CON	L	M	H
Average daily feed intake (g)	123.73 ± 6.65 ^a^	108.02 ± 3.79 ^c^	127.07 ± 3.98 ^a^	101.00 ± 6.94 ^c^
Average daily weight gain (g)	17.64 ± 0.96 ^c^	24.55 ± 1.12 ^a^	20.83 ± 1.19 ^b^	19.59 ± 0.80 ^b,c^
Feed conversion ratio (%)	7.14 ± 0.63 ^a^	4.44 ± 0.18 ^b^	6.27 ± 0.45 ^a^	5.05 ± 0.24 ^b^

Note: CON was the control group; L, M and H were 0.5%, 1% and 2% *P. glomerata* leaf addition groups, respectively. The results are expressed as mean ± SD, n = 5. Different letters on the inter-digit scale indicate significant differences (*p* < 0.05), lowercase letters indicate significance level a = 0.01, and the same letter or no letter indicates no significant difference (*p* > 0.05).

**Table 4:** Effects of *Pfaffia glomerata* leaves on the morphological structure of the duodenum, ileum and jejunum in New Zealand rabbits.

Treatment	Groups			
	CON	L	M	H
Duodenum				
Villus height (µm)	496.15 ± 14.45	507.04 ± 19.40	468.2 ± 30.43	514.37 ± 24.59
Crypt depth (µm)	112.71 ± 4.54 ^a^	91.00 ± 4.29 ^b^	90.7 ± 8.69 ^b^	95.78 ± 4.83 ^a,b^
Villus to crypt ratio	4.43 ± 0.18 ^a^	5.62 ± 0.27 ^b^	5.26 ± 0.35 ^a^	5.39 ± 0.18 ^a^
Jejunum				
Villus height (µm)	424.14 ± 30.85 ^b^	512.02 ± 20.61 ^a^	497.25 ± 27.81 ^a,b^	512.95 ± 20.77 ^a^
Crypt depth (µm)	109.05 ± 8.63	92.62 ± 3.02	99.70 ± 5.05	92.10 ± 7.16
Villus to crypt ratio	4.04 ± 0.64 ^b^	5.55 ± 0.22 ^a^	5.04 ± 0.38 ^a,b^	5.69 ± 0.55 ^a^
Ileum				
Villus height (µm)	405.05 ± 4.80 ^b^	521.57 ± 2.42 ^a^	434.42 ± 4.76 ^b^	512.39 ± 3.95 ^a^
Crypt depth (µm)	103.85 ± 21.73 ^b^	92.93 ± 14.54 ^b^	101.94 ± 16.39 ^b^	120.49 ± 2.08 ^a^
Villus to crypt ratio	3.93 ± 0.21 ^b^	5.63 ± 0.22 ^a^	4.29 ± 0.19 ^b^	4.28 ± 0.13 ^b^

Note: CON was the control group; L, M and H were 0.5%, 1% and 2% *P. glomerata* leaf addition groups, respectively. The results are expressed as mean ± SD, n = 5. Different letters on the inter-digit scale indicate significant differences (*p* < 0.05), lowercase letters indicate significance level a = 0.01, and the same letter or no letter indicates no significant difference (*p* > 0.05).

**Table 5:** Effects of *Pfaffia glomerata* leaves on the weight and index of immune organs in New Zealand rabbits.

Treatment	CON	CXT	L	M	H
Thymus weight (g)	1.34 ± 0.21 ^a^	1.03 ± 0.14 ^b^	1.47 ± 0.15 ^a^	1.67 ± 0.28 ^a^	1.38 ± 0.28 ^a^
Thymus index (%)	0.64 ± 0.17 ^a^	0.52 ± 0.10 ^a^	0.68 ± 0.13 ^a^	0.82 ± 0.21 ^b^	0.68 ± 0.10 ^b^
Spleen weight (g)	1.02 ± 0.10 ^a^	0.68 ± 0.10 ^b^	0.63 ± 0.07 ^b^	0.70 ± 0.05 ^b^	0.81 ± 0.01 ^b^
Spleen index (%)	0.49 ± 0.01 ^a^	0.31 ± 0.05 ^c^	0.32 ± 0.03 ^c^	0.31 ± 0.02 ^c^	0.41 ± 0.01 ^b^
Sacculus rotundus weight (g)	2.47 ± 0.12 ^b^	1.77 ± 0.18 ^a^	2.18 ± 0.21 ^b^	2.37 ± 0.20 ^b^	1.99 ± 0.24 ^b^
Sacculus rotundus index (%)	1.17 ± 0.11 ^b^	0.90 ± 0.15 ^a^	1.02 ± 0.05 ^b^	1.09 ± 0.12 ^b^	0.98 ± 0.09 ^a^

Note: CON: control group, CTX: immunosuppressive group, group L: 0.5% *P. glomerata* leaf addition group, group M: 1% *P. glomerata* leaf addition group, group H: 2% *P. glomerata* leaf addition group. The results are expressed as mean ± SD, n = 5. Different letters on the inter-digit scale indicate significant differences (*p* < 0.05), lowercase letters indicate significance level a = 0.01, and the same letter or no letter indicates no significant difference (*p* > 0.05).

**Table 6:** Effects of *Pfaffia glomerata* leaves on serum immunoglobulin (IgM and IgA) concentrations in New Zealand rabbits.

Treatment	Groups				
	CON	CXT	L	M	H
IgM (µg/mL)	5808 ± 229.10 ^c^	4453.48 ± 238.16 ^d^	6049.80 ± 134.22 ^b^	6625.82 ± 231.18 ^bc^	7820.91 ± 219.21 ^a^
IgA (µg/mL)	1425.98 ± 47.86 ^bc^	1361.05 ± 54.29 ^a^	1615.45 ± 47.62 ^ab^	1578.84 ± 102.27 ^bc^	1773.75 ± 60.44 ^a^

Note: CON: control group, CTX: immunosuppressive group, group L: 0.5% *P. glomerata* leaf addition group, group M: 1% *P. glomerata* leaf addition group, group H: 2% *P. glomerata* leaf addition group. The results are expressed as mean ± SD, n = 8. Different letters on the inter-digit scale indicate significant differences (*p* < 0.05), lowercase letters indicate significance level a = 0.01, and the same letter or no letter indicates no significant difference (*p* > 0.05).

**Table 7:** Horizontal species composition and relative abundance of microphyla in New Zealand rabbits.

Phylum (%)	Groups			
	CON	L	M	H
*Firmicutes*	59.18	56.65	60.08	64.94
*Bacteroidota*	30.38	32.63	26.58	25.65
*Synergistota*	3.93	3.34	4.21	3.26
*Verrucomicrobiota*	1.68	3.35	4.35	2.17
*Desulfobacterota*	1.21	1.70	2.19	1.40
*Actinobacteriota*	1.22	0.85	0.96	0.86
*Proteobacteria*	1.10	0.48	0.71	0.88
*unclassified_Bacteria*	0.46	0.38	0.41	0.35
*Patescibacteria*	0.47	0.42	0.28	0.34
*Spirochaetota*	0.12	0.12	0.13	0.05
*Cyanobacteria*	0.13	0.06	0.06	0.07
*Campylobacterota*	0.08	0.01	0.05	0.03
*Fusobacteriota*	0.01	0.00	0.00	0.00

Note: CON was the control group; L, M and H were 0.5%, 1% and 2% *Pfaffia glomerata* leaf addition groups, respectively.

**Table 8:** Species composition and relative abundance of microphyla in New Zealand rabbits.

Genus (%)	Groups			
	CON	L	M	H
*unclassified_Lachnospiraceae*	8.66	9.16	7.98	12.34
*Bacteroides*	12.96	8.10	7.09	6.13
*unclassified_Muribaculaceae*	8.16	11.66	6.79	5.62
*unclassified_Clostridia_vadinBB60_group*	8.69	5.52	7.32	7.60
*Christensenellaceae_R_7_group*	4.64	5.75	6.51	4.97
*Ruminococcus*	4.57	5.81	6.24	4.88
*NK4A214_group*	3.70	4.45	5.26	5.39
*dgA_11_gut_group*	1.63	4.68	3.35	4.49
*Akkermansia*	1.58	3.23	4.21	2.15
*Synergistes*	2.61	3.23	2.42	2.69
Others	42.79	38.39	42.63	43.75

Note: CON was the control group; L, M and H were 0.5%, 1% and 2% *Pfaffia glomerata* leaf addition groups, respectively.

## Data Availability

The original contributions presented in the study are included in the article, and further inquiries can be directed to the corresponding authors.
